# Using Probiotics as Supplementation for *Helicobacter pylori* Antibiotic Therapy

**DOI:** 10.3390/ijms21031136

**Published:** 2020-02-08

**Authors:** Jianfu Ji, Hong Yang

**Affiliations:** State Key Laboratory of Microbial Metabolism, School of Life Sciences and Biotechnology, Shanghai Jiao Tong University, Shanghai 201100, China; jijianfu@sjtu.edu.cn

**Keywords:** *Helicobacter pylori*, probiotic therapy, antibiotic therapy

## Abstract

*Helicobacter pylori* is a well-known pathogen that is highly prevalent in the world population, and *H. pylori* infection is potentially hazardous to humans because of its relationship to various gastrointestinal diseases, such as gastric ulcers, chronic gastritis, and gastric carcinoma. Therefore, the clinical guidelines recommend taking antibiotic therapy to eradicate the pathogen, which usually leads to the desired therapeutic effect. However, some failure cases of this therapy indicate that the increasing antibiotic resistance and side effects may affect the therapeutic effect. Here we propose that using probiotics as supplementation for antibiotic therapy may provide an extra help. Recent studies have shown that probiotic supplementation therapy has promising application prospects; it can enhance the antibiotic effect to achieve a better therapeutic result and maintain the balance of the host gastrointestinal microbiota. In summary, under global conditions of increasing *H. pylori* prevalence, probiotic supplementation therapy is worthy of further studies for future clinical application.

## 1. Introduction

*Helicobacter pylori* is a microaerobic, spiral, flagellated Gram-negative pathogen that has colonized approximately 50% of the world’s population yet, the infection rate in China has exceeded 80% and may continue to increase in the future [1,2]. Once *H. pylori* successfully colonized the stomach, it evolves toward persistent chronic infection with spontaneous clearance being relatively rare [3]. Although the majority of infected individuals are clinically asymptomatic, the host can develop gastric ulcers, chronic gastritis or other gastrointestinal diseases, 1–3% of *H. pylori*-infected people are at risk of developing gastric cancer [4]. Thus, medical guidelines recommend antibiotic therapy as a good option for clinical eradication of *H. pylori* [5]. Recent studies have shown that the eradication of *H. pylori* in infected asymptomatic individuals at all ages can reduce the occurrence of gastric cancer [6]. However, failure cases in this antibiotic therapy indicate that drug-resistant strains and side effects may occur in some patients, which can affect the treatment effect [2,7].

Probiotic supplementation therapy is an emerging therapy for *H. pylori* treatment [8]. Probiotics are defined as live microorganisms which, when administered in adequate amounts, confer a health benefit on the host [9]. Probiotics have natural advantages, such as safety, immunomodulation, and anti-pathogen ability, and are often used to treat gastrointestinal diseases alone or in combination with drugs [10,11,12]. Most probiotics are deemed to colonize the human gut, and certain species, such as *Lactobacillus* spp., can colonize the human stomach, directly or indirectly antagonizing *H. pylori* [13,14,15]. It has been reported that taking probiotics alone can diminish bacterial load, whereas using probiotics along with antibiotics can improve the eradication rate and alleviate side effects [16,17]. Using probiotics to treat *H. pylori* infection is a feasible way but it shows some uncertainty. The optimal dose, the time of dosing, the duration of therapy, and the interaction mechanisms among the selected probiotics and antibiotics remain to be explored [8].

This review summarizes recent studies about the *H. pylori* infection process, its antibiotic therapy, mechanisms of probiotic therapy, and clinical studies. We highlight the advantages of using probiotics in combination with antibiotics for enhancing antibiotic drug efficacy and restoring the gastrointestinal microbiota. Specific strains can be supplemented during *H. pylori* clinical treatment to achieve a better antibiotic therapy efficacy. Therefore, this review is instructive for the *H. pylori* clinical eradication through developing probiotics as an alternative therapy. It is worth noting that the effects of probiotic therapy vary greatly because of strain specificity.

## 2. *H. pylori* Infection and Antibiotic Therapy

### 2.1. Colonization Mechanisms

**Figure 1:**
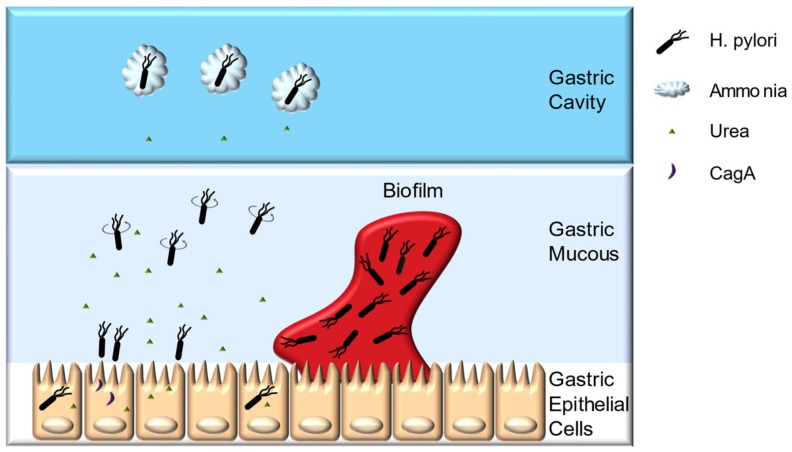
Colonization of the stomach by *H. pylori*.

*H. pylori* can cause diseases only if successfully colonized. The optimal growth pH for *H. pylori* is 8.5, whereas the bacteria can survive for only approximately 30 min under extremely acidic environments, such as gastric cavity [18,19]. The ability of *H. pylori* to transiently resist gastric acid and pass through the gastric mucous layer, quickly reaching the pH-neutral environment, mainly depends on its urease, chemotaxis system, flagella, and spiral morphology (Figure 1) [20]. In addition, the capacity for gastric epithelial cell adherence, biofilm formation, and antioxidant enzyme system help *H. pylori* achieve long-term colonization [20,21,22,23,24].

*H. pylori* urease accounts for approximately 10% of its total protein mass, playing a pivotal role in both establishing initial colonization and maintaining chronic infection [25,26]. Urease can hydrolyze urea to produce carbon dioxide and ammonia, and the latter being able to buffer the gastric acids around the bacteria to maintain its viability [26]. Furthermore, the morphology of the gastric mucin is closely related to the pH value. Gastric mucin forms a gel under low pH, whereas the increase in pH caused by urease catalysis loosens gastric mucin, enabling *H. pylori* to swim more easily [20].

The chemotaxis system, flagella, and spiral morphology of *H. pylori* enable its swift passage through the gastric mucous layer. Urea is not only the substrate of urease, but also one of the signaling molecules of this chemotaxis system. *H. pylori* uses chemotaxis system to sense the pH gradient, urea and amino acids secreted by the host cells to position itself [25,27]. Meanwhile, the chemotaxis system is capable of receiving adversity signals such as reactive oxygen species (ROS), bile salts emitted by host cells, and quorum sensing molecules (autoinducer-2, AI-2) produced by the bacteria itself, thereby driving *H. pylori* away from the harsh environment [28]. Chemotaxis system integrity is indispensable for *H. pylori* pathogenesis. McGee et al. reported that the CheY chemotaxis regulator deficient *H. pylori* strain cannot colonize Mongolian gerbils, and the TlpB chemoreceptor-deficient strain can infect Mongolian gerbils with a substantial decrease in inflammation [29]. The flagella and spiral morphology of *H. pylori* facilitate its passage through the gastric mucous layer. *H. pylori* has polar flagella with rotation that is powered by the motor proteins MotA and MotB, and mutants with incomplete flagella has a reduced ability to infect mice gastric, whereas mutants with more flagella number can swim faster through simulated gastric mucous layer [30,31]. Spiral morphology enables *H. pylori* to drill through the gastric mucous layer like a rotating cork, mutants with straight-rod morphology will lose about 7–21% of its swimming speed [25,31].

After passing through the gastric mucous layer, *H. pylori* can persistently colonize the space between the gastric mucous layer and gastric epithelial cells, mainly relies on its abilities of epithelial cell adherence and invasion, biofilm formation, and its antioxidant enzyme system. *H. pylori* encode approximately 30 outer membrane proteins (OMPs), such as blood group antigen-binding adhesion (BabA), and sialic acid-binding adhesion (SabA). These OMPs can bind to the recognition sites on gastric epithelial cells, providing a basis for long-term colonization [25]. Successfully colonized *H. pylori* can invade host epithelial cells and immune cells under unfavorable conditions [32]. It has been confirmed that *H. pylori* can invade and proliferate in gastric epithelial cells such as AGS and MKN45 cells, which may enable them to avoid the host immune system [32]. Moreover, *H. pylori* can form biofilms in gastric mucosa, and biofilm formation might enhance its resistance to drugs and the host immune system [21,22,23,24,33]. In vitro experiments have shown that the resistance of *H. pylori* ATCC43629 biofilm cells to clarithromycin is approximately eight-fold greater than that of planktonic cells [34]. Additionally, biofilm formation increases *H. pylori* TK1402 generation rates of drug-resistance mutations and its resistance to metronidazole, clarithromycin, and amoxicillin in vitro [22,24,35]. The antioxidant system is another defense mechanism of *H. pylori*. These bacteria resist the oxidative stress generated by the host immune response by invoking its own antioxidant enzymes, such as superoxide dismutase (SOD) and hydrogen peroxide (CAT) [36,37,38]. The infection rate of the SOD-deficient strain in mice is only 4%, whereas for the wild-type strain is as high as 88%, indicating the importance of antioxidant enzymes in *H. pylori* infection [39].

### 2.2. H. pylori Pathogenesis

Although about 85% of colonized individuals are asymptomatic or have mild gastritis, 15% of infected people still have a chance to develop peptic ulcer disease (PUD) during the long-term *H. pylori* infection, and about 1% can develop gastric cancers [40]. After successful colonization of the stomach, these bacteria can activate host’s innate and adaptive immune response, which may induce atrophic gastritis, dysplasia, metaplasia, and ultimately gastric carcinoma [41]. Recent studies have shown that the eradication of *H. pylori* in infected asymptomatic individuals at all ages can reduce the occurrence of gastric cancers [6]. Various virulence factors and virulence genes of *H. pylori*, such as cytotoxin-associated gene A protein (CagA), vacuolating cytotoxin protein (VacA), duodenal ulcer promoting gene (DupA), and urease play the key role in injuring host tissues and inducing gastrointestinal diseases [19].

CagA is an oncogenic protein encoded by *cagA* gene located at the end of cag pathogenic island (cagPAI), it is closely related to the occurrence of gastric adenocarcinomas and PUD [42]. After being injected into host cells through a type IV secretion system, CagA can interact with multiple host cell molecules, thus altering the intracellular signal transduction pathways of gastric epithelial cells to induce pro-inflammatory responses, leading to chronic inflammation of gastric mucosa. Meanwhile, CagA can facilitate carcinogenesis through the modulation of apoptosis, disruption of cell polarity, and promotion of genetic instability [43]. Apoptosis of gastric epithelial cells causes a decrease in gastric acid secretion and creates a more conducive environment for the *H. pylori* growth [44]. Studies show that individuals infected with *cagA*-positive *H. pylori* strains are associated with two-fold higher risk of distal gastric cancers and 1.69-fold risk of PUD compared to those infected with *cagA*-negative strains [40]. 

VacA toxin exists in most *H. pylori* strains, but varies greatly in its toxin activity. It is named after its ability to form “vacuoles” in gastric epithelial cell cytoplasm [19,45]. VacA have been described as a multi-receptor protein with receptors being found on the surface of various cells, including gastric epithelial cells, and immune cells [46]. After binding to these receptors, VacA can enter the host cells and accumulate inside different cellular compartment to induce membrane depolarization, mitochondrial dysfunction, autophagy, activation of mitogen-activated protein kinases, inhibition of T cell function, and cell apoptosis [42,47]. Studies show that individuals infected with *H. pylori* harboring *vacA* s1 or m1 have an increased risk of gastric cancer in Western populations, while *vacA* i1 type *H. pylori* infection is associated with higher gastric cancer risk in the Middle Asia and Middle East area [40].

DupA is named for its ability to increase the occurrence of duodenal ulcer (DU) [40]. The prevalence of this gene was higher in strains from patients with duodenal ulcer than in those with gastritis or gastric cancer [48]. Data analysis have shown that individuals carrying *dupA*-positive strains are under increased risk for duodenal ulcers and gastric cancers [43].

In addition, *H. pylori* urease and its catalytic products may cause direct damage to host tissues. Ammonium ions can destroy the integrity of the connection between gastric epithelial cells, while carbon dioxide supports bacteria resistance to damage from nitric oxide metabolites and peroxynitrite produced by phagocytic cells [26]. Urease also induces inflammation and angiogenesis in vivo independently of its catalytic activity and directly activates human neutrophils to produce reactive oxygen species, thereby injuring the host body [49].

### 2.3. Antibiotic Therapy

Antibiotic therapy is the most effective and widely used treatment for *H. pylori* infection. Antibiotics with significant anti-*H. pylori* effects in vitro tends to be unstable in the extremely acidic environment in human stomach, thus being difficult to maintain high concentrations to kill the bacteria, leading to a poor clinical effect [50]. Antibiotics with good acid resistance and bactericidal effects, such as metronidazole, clarithromycin, and amoxicillin, are the most commonly used drugs in *H. pylori* clinical therapy [51]. In addition, one antibiotic alone cannot eradicate the bacteria thoroughly, therefore multiple drug therapies are recommended [52]. Most commonly used antibiotic therapy, triple therapy, is based on the combination of two antibiotics plus one proton pump inhibitor (PPI), with a duration of 7–14 days [50]. The antibiotics play a major role in eradicating the pathogen, while the PPI acts to inhibit gastric acid secretion and thus enhance the effect of the antibiotics [53].

Triple therapy was considered the most effective therapy for *H. pylori* eradication in the past, however the increase in drug-resistant strains over the years has changed this situation [54,55]. It has been reported that the resistance rate of *H. pylori* to metronidazole in China increased by approximately 50% between 2000 and 2014, and clarithromycin resistance has increased from approximately 14.8% in 2000 to approximately 52.6% in 2014 [2]. Traditional triple therapy based on metronidazole and clarithromycin tends to have an eradication rate below 80%, which no longer meets the clinical need [8]. The increase in antibiotic-resistance strains poses a severe challenge for *H. pylori* treatment, and therefore, enhancing the efficacy of old antibiotics or developing new antibiotics is currently considered feasible solutions.

Another challenge for the antibiotic therapy of *H. pylori* are side effects. These may occur in some patients, the combination of two antibiotics causes more damage to the host gastrointestinal microbiota and leads to the imbalanced microbial population associated with the proliferation of gastrointestinal pathogens [56,57,58]. Additionally, PPI induction decreases in gastric acid secretion may increase the mobility of these pathogens, enable them to circulate in the gastrointestinal tract [59,60]. Side effects occur in those patients may cause a decline in treatment efficiency and make retreatment difficult.

## 3. Probiotic Therapy

### 3.1. Antagonistic Mechanism

**Figure 2:**
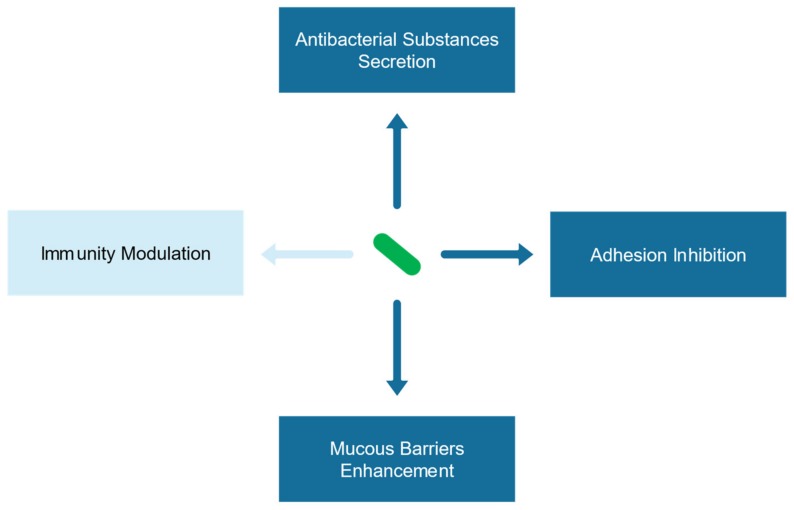
Antagonistic mechanism of probiotic against *H. pylori*.

As an emerging adjuvant, probiotics have been used to treat a series of gastrointestinal diseases, including *H. pylori* infection. In vitro experiments demonstrated that various probiotics have the potential to antagonize *H. pylori* through their metabolites or bacterial cells [61,62,63]. Sun et al. found four *Lactobacillus* strains isolated from fermented foods in northeastern China were able to inhibit the growth of *H. pylori* [63]. The acid-resistant strain *L. johnsonii* No.1088, isolated from gastric juice of healthy volunteers could suppress *H. pylori* both in vitro and in a mouse model, and the heat-killed form of the strain also showed antibacterial effects [64,65]. The antagonism of probiotics against *H. pylori* is achieved through a series of direct or indirect interactions, including secreting antibacterial substances, competing inhibition, enhancing mucous barriers, and regulating immunity (Figure 2) [41].

Probiotics can secrete antibacterial substances such as lactic acid, short-chain fatty acids (SCFAs), hydrogen peroxide, and bacteriocin [16]. Lactic acid and short-chain fatty acids normally show more intensive antibacterial ability than strong acids because of their incomplete ionization, the undissociated form of these organic acids can damage *H. pylori* cells by functioning as proton carriers that induce acidification of the cytoplasm and the accumulation of toxic anions [66]. Zheng et al. conducted an in vitro study on *L. pentosus* LPS16, and found that lactic acid can inhibit both drug-sensitive and drug-resistant *H. pylori* strains [67]. Meanwhile, lactic acid can suppress *H. pylori* urease activity [68]. In addition to organic acids, hydrogen peroxide produced by probiotics can cause oxidative damage to pathogenic proteins, membrane lipids and DNA by forming peroxygen ions, thus injuring the *H. pylori* cell [69]. Furthermore, certain probiotics can produce bacteriocins that have a direct antibacterial effect on *H. pylori*. Most bacteriocins are thermostable peptides with antagonistic activity against planktonic cells and/or biofilm cells [70]. Among seven bacteriocins derived from lactic acid bacteria, lacticin A164 and BH5 secreted by *Lactococcus lactis* showed the greatest effectiveness against *H. pylori* ATCC43504 and DSM [71]. Boyanova et al. found that the bacteriocin secreted by seven *L. bulgaricus* strains not only inhibited the growth of antibiotic-sensitive *H. pylori* strains, but also the antibiotic-resistant strains [72]. In addition to these peptide-like bacteriocins, a nonpeptide antipathogen substance synthesized by *L. reuteri*, called reuterin, can inhibit *H. pylori* growth and downregulate the expression of the virulence genes *vacA* and *flaA* [73].

Probiotics may hinder *H. pylori* colonization by competing for binding sites or disturbing the adhesion process. Probiotics with high affinity for epithelial cells can block the colonization of pathogenic bacteria in gastrointestinal epithelial cells. It has been shown that *L. reuteri* JCM1081, TM105 can compete with *H. pylori* for the asialo-GM1 and sulfatide binding sites in gastric epithelial cells, thereby inhibiting early *H. pylori* colonization [74]. Two *L. gasseri* strains can affect *H. pylori* colonization by inhibiting the expression of *H. pylori* adhesion gene *sabA* [75]. *Saccharomyces boulardii* has neuraminidase activity selective for α(2,3)-linked sialic acid of host cell, thus removes *H. pylori* binding sites [76].

The enhancement of the mucous barrier by probiotics helps the host to hinder *H. pylori* colonization. *H. pylori* infection can downregulate the expression of *muc1* and *muc5AC* gene in KATO III cells, which may cause mucous layer disruption in vivo [77]. Probiotics can upregulate tight-junction proteins and promote the mucous secretion by increasing the expression of *muc1*, *muc2*, and *muc3*, thus stabilizing the mucous layer [78]. These properties indicate that the host body may better resist *H. pylori* invasion by relying probiotics on repairing the gastric mucosal barrier, effectively preventing the initial infection and reinfection of the pathogen.

In addition to these nonimmune effects, probiotics can alleviate the host inflammation caused by *H. pylori* infection. *H. pylori* infection-induced inflammatory diseases are associated with the sustained expression of inflammatory factors, and these factors do not eliminate *H. pylori* but lead to the continuation of the inflammatory response [79]. Probiotics can inhibit pro-inflammatory factor expression, thereby mitigating the inflammatory response. Numerous studies have shown that probiotic strains such as *L. acidophilus*, *L. bulgaricus*, and *L. rhamnosus* can reduce the expression of IL-8 in *H. pylori*-infected cells [17,61,80]. Yang et al. demonstrated that although *H. pylori* infection causes the overexpression of IL-8, TNF-α and other pro-inflammatory factors in MKN45 cells, pretreatment with high doses of *L. acidophilus* La5 can silence the Smad7 and NF-κB pathways, thus relieving the inflammatory response [79]. Therefore, probiotics have a preventive and mitigating effect on the inflammation caused by *H. pylori* infection.

### 3.2. Clinical Studies

The use of probiotics alone or in combination with antibiotics has been reported in many clinical trials. Specific probiotic therapy not only improves the eradication rate but also reduces the side effects caused by antibiotic therapy [81]. Gotteland et al. categorized 182 asymptomatic children with *H. pylori* infection into four groups, namely, the triple therapy group, *S. boulardii* plus inulin group, *L. acidophilus* LB group, and drug-free group. The results showed that the level of *H. pylori* in infected children in the *S. boulardii* plus inulin group reduced, and that as many as 12% of the children achieved pathogen eradication [82]. Another study claimed that *L. reuteri* combined with PPI could provide approximately 12.5% eradication rate without the use of antibiotics [9]. Although the use of probiotics alone has some effect, this approach does not meet the clinical needs, and use of probiotics as auxiliary in antibiotic therapy achieves greater significant effects [51]. Fonseca et al. analyzed the results from ten clinical trials involving antibiotic therapy supplemented with different *L. reuteri* strains and all the antibiotic-probiotic combination groups exerted greater eradication effects than was realized with the single antibiotic group, with the exception of one study [83]. Sýkora et al. treated 86 children with *H. pylori* infection and found that the eradication rate from the use of *L. casei* DN114001 along with triple therapy was 84.6%, while for the triple therapy group alone, it was 57.5%, and probiotic supplementation reduced the severity of the side effects simultaneously [84]. Probiotic supplementation therapy can improve not only the eradication rate of antibiotic-sensitive *H. pylori* strains but also that of the resistant strains. For clarithromycin-resistant strains, use of *L. lactis* OLL2716 in combination with triple therapy increased the eradication rate by approximately 10% compared to that of the single triple therapy [85]. Furthermore, fermented milk preparation containing multiple probiotics also improves the eradication rate of triple therapy by 5–15% [86].

However, some reports claim that the use of probiotics in clinical treatment exerts no significant effects [87]. We deem these failure may be related to the selected probiotic species and/or antibiotic class. Only the correct probiotic strain applied in the appropriate manner will lead to therapy that achieves the desired therapeutic effect.

## 4. Advantages of Probiotic Supplementation Therapy

### 4.1. Drug Synergy and Mutant Prevention Theories

The range of drug concentrations for which drug-resistant mutants are most readily induced is called the mutant selection window and it extends from the minimum inhibitory concentration (MIC) to the mutant prevention concentration (MPC) [88]. When the drug concentration is within the mutant selection window, the growth of drug-sensitive strains is inhibited, whereas the proportion of resistant strains increases [89]. Although taking a low dose of drugs does not lead to a high incidence of resistant strains, it does not achieve good clinical effects, and increasing the dose might eradicate the pathogen with aggravating side effects. Multiple drug combinations can better resolve these issues [90,91,92,93]. According to drug synergy theory, multiple drugs used together do not yield a simple effect of 1 + 1 = 2; they may have a synergistic or antagonistic effect [92]. Synergistic effects can improve drug efficacy and prevent the emergence of drug-resistant bacteria because of the difficulty in producing multiple drug mutants [93]. Thus, using synergistic drugs for *H. pylori* treatment can decrease MPC and minimize the mutant selection window to achieve better efficacy and prevent an increase in drug-resistant bacteria. The synergistic effect is not limited to antibiotics, antibiotics and non-antibiotic adjuvants also have synergistic effects [90].

Although most probiotics are localized in the gut, certain probiotics can colonize the pH-neural part of the stomach [13,14]. Substances metabolized by these strains may have the potential to act synergistically with antibiotics. In vitro experiments have shown that the combination of tetracycline and probiotic fermentation broth has greater antimicrobial effects against *Pseudomonas aeruginosa* clinical resistant strains than either tetracycline or probiotic fermentation broth does alone [94]. Yang et al. conducted an antibacterial study on *Clostridium difficile*, and the results showed that *Bifidobacterium breve* YH68 cell-free supernatant can enhance the synergistic effect of antibiotics and weaken the antagonistic effect [95]. Consequently, a combination of probiotic metabolites and antibiotics can have a greater antibacterial effect against either Gram-negative or Gram-positive pathogens. Although there are a few in vitro studies on *H. pylori* to confirm the synergy between probiotics and antibiotics, the positive clinical results hint at a potential interaction.

### 4.2. Biofilm Theory

Biofilms are bacterial populations that adhere to each other or the surface of a medium, containing viscous and protective substrates, and can thus transfer nutrients to internal bacteria and excrete detrimental metabolites by forming channels to improve bacterial resistance to hostile environments [96]. Recent studies confirmed that *H. pylori* has the ability to form biofilms both in vitro and in vivo and the biofilm formation may play a pivotal role in long-term colonization and related gastrointestinal diseases [21,97]. Coticchia et al. analyzed the gastric mucosa of *H. pylori* urease-positive and urease-negative populations, and the results revealed that the average coverage of the biofilms in the gastric mucosa was 97.3% in the urease-positive patients, while in the urease-negative patients, it was only 1.64% [33]. These results suggest the prevalence of *H. pylori* biofilms in the human gastric mucosa. Moreover, in vitro experiments showed that *H. pylori* clinical strains TK1402 and TK1049 could increase the tolerance to clarithromycin, amoxicillin, and metronidazole after biofilm formation [24]. The drug-tolerance conferred by biofilms may depend on the overexpression of drug efflux pumps or an increased rate of antibiotic resistance mutations [23,35,98]. The formation of *H. pylori* biofilms may also create defenses against the host immune system [21,99,100].

Considering *H. pylori* biofilms and the greater antibiotic resistance they confer, destroying the biofilm to enable the administration of a reduced antibiotic dose is a new strategy for *H. pylori* treatment [33]. Cammarota et al. separated 40 infected patients with *H. pylori* eradication failure into two groups for retreatment. The control group was given antibiotic therapy only, and the experimental group was given the anti-biofilm agent *N*-acetylcysteine before antibiotic therapy. The cure rate of the experimental group was 65%, while that the control group was only 20%, and the biofilm was found only in the infected individuals with failed eradicate again upon retreatment [101]. The results suggest that the use of an anti-biofilm preparation along with antibiotics may result in better clinical treatments. Although there are few direct reports of probiotics use against *H. pylori* biofilm, reports have confirmed that probiotics can destroy the mature biofilms of pathogenic bacteria, such as *Enterococcus faecalis* and *Staphylococcus aureus* [102,103,104,105]. In addition, the bacteriocin secreted by *L. brevis* can inhibit *Escherichia coli* and *Salmonella typhimurium* biofilm formation [70]. These findings indicate that probiotics are likely to have a killing effect on *H. pylori* biofilm, and the destruction of these biofilms may improve the effect of the antibiotics, thereby achieving better eradication effects while reducing the clinical dose of antibiotics necessary.

### 4.3. Gastrointestinal Theory

The human gastrointestinal microbiota theory suggests that gastrointestinal microbes and their metabolites can modulate human physiological activities, such as nutrient absorption, energy metabolism and immune function, and the bacteria, host, and environment are interdependent and mutually constrained in a dynamic balance [15,106,107,108,109]. *H. pylori* infection alters both the gastric and intestinal microflora [110]. At the same time, the bactericidal effect of antibiotic use and the pH change caused by PPI can lead to more severe disorders [56,57,58,59,60]. Clinical studies have confirmed that *H. pylori* infection reduces the diversity of the stomach microbiota in children and adults, and this microbiota may not be restored after eradicating the pathogen [15,111]. The evidence from an analysis of the gastric microbiota of infected people and noninfected people revealed that the abundance of *Proteobacteria* and *Spirochetes* in the former is higher than that of the latter [15]. In mouse model, *H. pylori* infection changes the intestinal relative microbial abundance in an indirect way based on the fact that the bacteria has not been detected in mice feces [110]. Furthermore, antibiotic eradication therapy create additional changes in the gastrointestinal microbiota. *Lactobacillus* spp.in the stomach dramatically decreased after penicillin was administered to a mouse model [15]. In an infected population treated with quadruple therapy, the alpha diversity and *B. adolescentis* abundance in the gut microbiota was significantly decreased, and some *E. faecalis* strains acquired even greater antibiotic resistance [57]. Intake of probiotics may produce positive effects in terms of protecting and recovering the gastrointestinal microbiota [112].

Experiments with animal models and clinical trials have shown positive effects of probiotics on the host gastrointestinal microbiota. After treatment with *L. rhamnosus* GMNL-74 or *L. acidophilus* GMNL-185, the abundance of *Bifidobacterium* spp. and *Akkermansia muciniphilia* in *H. pylori*-infected mice was significantly increased [61]. Wu et al. found that the diversity of the gut microbiota is remarkably reduced when *H. pylori* infected individuals are treated with triple therapy alone, while supplementation with *Bacillus subtilis* and *E. faecalis* can inhibit this reduction [56]. Moreover, colonization of specific probiotics in gastric likely maintain the balance of the gastric microbiota [15]. Therefore, probiotic supplementation is effective for maintaining both the gastric and gut microflora during *H. pylori* infection and antibiotic therapy.

## 5. Outlook

Because of the high prevalence of *H. pylori* infection and its correlation with gastrointestinal diseases, eradicating the pathogen is certainly a good solution for clinical treatment [5,113]. The current clinical use of antibiotic-related therapies is a great option but may be insufficient in some patients. Probiotic supplementation therapy has been clinically proven to enhance the efficacy of antibiotics, maintain the host gastrointestinal microflora, and reduce side effects. However, it is difficult to determine which probiotics are most effective in *H. pylori* treatment based on the existing studies, and different combinations of probiotics and antibiotics can produce unparallel effects. Therefore, relevant experiments should be conducted as theoretical support to explore the selection and appropriate dosage of probiotics and antibiotics to enhance antibiotic efficacy. In addition, the synergistic effect of probiotics and antibiotics should be leveraged not only to target the planktonic bacteria cells but also to induce a corresponding effect on the stubborn biofilm cells and thus prevent repeated infection. Finding the optimal combination of probiotics and antibiotics will promote the effectiveness of the first-line treatment of *H. pylori* by reducing the dosage of the antibiotics needed and the occurrence of side effects during treatment, thus achieving the maximum killing effect on this pathogen.
